# MXene Based Flame Retardant and Electrically Conductive Polymer Coatings

**DOI:** 10.3390/polym16172461

**Published:** 2024-08-29

**Authors:** Bo Lin, Ao Li, Ivan Miguel De Cachinho Cordeiro, Ming Jia, Yuan Xien Lee, Anthony Chun Yin Yuen, Cheng Wang, Wei Wang, Guan Heng Yeoh

**Affiliations:** 1 School of Mechanical and Manufacturing Engineering, University of New South Wales, Sydney, NSW 2052, Australia; bo.lin@unsw.edu.au (B.L.); ao.li@unsw.edu.au (A.L.); i.decachinhocordeiro@unsw.edu.au (I.M.D.C.C.); xien.lee@student.unsw.edu.au (Y.X.L.); c.wang@unsw.edu.au (C.W.); g.yeoh@unsw.edu.au (G.H.Y.); 2 Urban Mobility Institute, College of Transportation Engineering, Tongji University, 4800 Cao’an Rd, Shanghai 201804, China; jiamingtj@tongji.edu.cn; 3 Key Laboratory of Road and Traffic Engineering of the Ministry of Education, College of Transportation Engineering, Tongji University, 4800 Cao’an Rd, Shanghai 201804, China; 4 Department of Building Environment and Energy Engineering, The Hong Kong Polytechnic University, Hung Hom, Kowloon, Hong Kong SAR, China; anthony-cy.yuen@polyu.edu.hk; 5 Australian Nuclear Science and Technology Organisation (ANSTO), Kirrawee DC, NSW 2232, Australia

**Keywords:** MXene, flame retardant, electro-magnetic interference (EMI) shielding, polymer coating

## Abstract

Modern polymer coatings possess tremendous multifunctionalities and have attracted immense research interest in recent decades. However, with the expeditious development of technologies and industries, there is a vast demand for the flame retardancy and electrical conductivity of engineered polymer coatings. Traditional functional materials that render the polymer coatings with these properties require a sophisticated fabrication process, and their high mass gains can be a critical issue for weight-sensitive applications. In recent years, massive research has been conducted on a newly emerged two-dimensional (2D) nanosize material family, MXene. Due to the excellent electrical conductivity, flame retardancy, and lightweightness, investigations have been launched to synthesise MXene-based polymer coatings. Consequently, we performed a step-by-step review of MXene-involved polymer coatings, from solely attaching MXene to the substrate surface to the multilayered coating of modified MXene with other components. This review examines the performances of the fire safety enhancement and electrical conductivity as well as the feasibility of the manufacturing procedures of the as-prepared polymer composites. Additionally, the fabricated polymer coatings’ dual property mechanisms are well-demonstrated. Finally, the prospect of MXene participating in polymer coatings to render flame retardancy and electrical conductivity is forecasted.

## 1. Introduction

Functional polymer coatings that render the substrates with exceptional properties such as flame retardancy [1], electrical conductivity [2,3], anti-corrosion [4,5], antimicrobial, waterproof [6,7], etc., have been adopted worldwide and occupy every corner of humans’ daily lives [8]. Nevertheless, with the rapid development of some emerging industries, such as hydrogen energy, oil/gas, high-end manufacturing, and telecommunication technologies, traditional polymer coatings cannot meet the constantly growing requirements and standards, especially for the points of flame retardancy and electrical conductivity. The electrical conductivity of the polymer coatings may help dissipate the static electricity and thus avoid electrostatic-induced fire accidents, which is of great significance in hazardous industries, such as the aeronautical industry [9], oil/gas and mining industries [10], electronics [11], and manufacturing industries [12]. In addition, with excellent flame-retardancy properties, polymer coatings will simultaneously protect the substrates from being easily ignited, mitigate the fire spread, and reduce the fire hazards in any potential fire scenarios.

To date, there is reportedly a huge global demand for flame retardant and electrically conductive polymer coatings [13]. Nevertheless, current challenges lie in the traditional technologies and functional coating materials being unable to satisfy the stricter standards and higher requirements of the product qualities. The current mainstream solutions in both academic and industrial areas are mainly the utilisation of metal particles [14], polypyrroles [15], and polyanilines [16]. Although these conductive materials may endow polymer coatings with good electrical conductivity and acceptable fire-resistance performances, some additives, such as conductive metals, may simultaneously increase the weight gain. Consequently, the applications of these materials in weight-sensitive electrical equipment are highly constricted. Additionally, during the combustion of the coated composites, the polymer coatings or the substrates may generate toxic and asphyxiant gases in the final volatiles, and the techniques for synthesising the coating materials and fabricating the coated polymers can be sophisticated [17]. Hence, considering the disadvantages to human health, environmental protection, cost-effectiveness and performance, the common materials shown above are restrictively employed in many areas due to the increased high constraints. Therefore, to cope with the current situation, fabricating polymer coatings with high electrical conductivity and flame-retardant performances is of great significance in advancing the development of multifunctional polymer coatings.

In 2011, Gogotsi and his colleagues synthesised and discovered the family of two-dimensional (2D) transitional metal carbides, nitrides, and carbonitrides, namely MXene [18]. Through etching the “Al” layer from the MAX phase Ti_3_AlC_2_ precursor (where “M” represents the early transitional metal, “A” means the third or fourth main group element, and “X” is the carbon or nitrogen element) using hydrofluoric acid, the layered Ti_3_C_2_ 2D nanosheets were fabricated for the very first time [18,19]. Since then, intensive investigations have been launched on MXene family materials and their applications. Owing to its 2D structure being similar to graphene, MXene can be applied to tremendous areas due to its structural, physical and chemical properties. It has been reported that MXene possessed intrinsic lightweight, electrical conductivity, visible and UV light absorption, thermal conductivity, etc., and showed excellent performances in energy storage, hydrogen evolution reactions (HER) catalysts, sewage treatment, fire safety and other areas. According to the scholars, MXene possesses high metallic conductivity due to the free electrons of the transition metal carbide or nitride backbones, especially the d-electrons from the transition metals [20]. Moreover, it should be pointed out that for the ordered double-transition metal structures, the outer metal layers play more important roles than the inner metal layers in terms of electrical properties. Additionally, studies also found that the proposed mechanisms of the MXene’s flame-retardant performances can be categorised into three sections: (1) Excellent heat propagation behaviour along the surface of the nanosheets to avoid heat penetration to the underneath materials [21]; (2) Superior physical barrier effect due to the formed dense TiO_2_ (take Ti_3_C_2_T_x_ nanosheets as an example) during combustion, which further prevents the heat transfer and isolates the generated combustible violates from reacting with oxygen in the outside atmosphere [22,23]; (3) Layer-stacked structure inside the polymer composites, which prolong pathways of the produced gaseous volatiles [24]. Consequently, with the properties mentioned above, MXene has proven to be one of the most suitable candidates for endowing polymer composites, films and membranes with different functions, especially for electrical conductivity and fire resistance.

Likewise, the principles introducing MXene in polymer composites, films, and membranes are also deployed to polymer coatings, with the purpose of attaining exceptional electrical conductivity and flame retardancy via the utilisation of MXene and its derivatives. It should be noted that, although tremendous research works were conducted on MXene, it was only until recent years, the applications of using MXene for rendering a material with both flame retardancy and high electrical conductivity as a polymer coating were carried out. Gogotsi and his colleagues first explore the feasibility of using MXene film and nanocomposites as electrodes for supercapacitors due to MXene’s superior electrical conductivity [25]. After that, researchers started to investigate the potential of using MXene as a coating material to endow substrate with electrical conductivity properties [26]. For the flame-retardant performance, it was until 2018, academics realised the possibility of using MXene to introduce fire safety performances [27]. In the following years, studies on using MXene as a coating material to provide substrates with flame-resistance properties were carried out [23]. Later, MXene-based polymer coatings have been widely reported, incorporating various methods to effectively enhance the electrical conductivity and flame retardancy of polymer coatings and be applied in various fields. Research works on using MXene for both flame retardancy and electrical conductivity were conducted [28]. Additionally, only until recent years, studies focusing on the utilisation of MXene as a coating to render the substrates with both fire resistance and enhanced electrical conductivity were performed [29].

This review summarises recent works that employed MXene as a critical coating component to improve polymer coatings’ electrical conductivity and fire safety performances to fulfil current and future industry needs. Statistical results indicate that since 2020, a growing corps of researchers has been working on exploring both the electrical conductivity and flame-retardancy performances of the MXene coatings. To gain a clear understanding of the mechanisms that allow MXene to render a substrate with these desired properties, we first reviewed the studies that used only unmodified MXene as coatings. This was conducted so that the fire resistant and electrical conductivity mechanism of MXene can be revealed. We then reviewed the articles that deployed only modified MXene as coatings. Consequently, methods on avoiding some drawbacks of pure MXene are discussed and new mechanisms can be found. Next, we included articles using coating combinations of unmodified MXene and other components. In this case, multifunctional coatings are achieved, and possible synergistic effects can be discovered. Finally, we discussed the studies combining modified MXene and other components as coatings. Therefore, a clear development of approaches on amending MXene disadvantages, functionalising MXene, rendering materials with multiple functions, and analysing the underneath mechanisms involved can be understood. We comprehensively analysed and compared the dual properties of the current findings and tried to figure out the optimised material compositions and feasible methods that can be employed to work together with MXene to realise outstanding flame retardancy and electrical conductivity. This review concludes the state-of-the-art development of MXene-based polymer coatings and is anticipated to summarise and provide theoretical and practical strategies for advancing the research and development of polymers with flame retardancy and electrical conductivity features.

## 2. Methodologies for Characteristic Performance

In order to better understand the enhanced fire resistance, electrical conductivity properties and structures of the as-prepared products, it is of importance to clarify the current testing technologies and approaches deployed. This section is divided into three parts that briefly describe the methods to evaluate the materials’ characterisation, flame retardancy and electrical conductivity.

### 2.1. Material Characterisation

In general, traditional methods for examining the quality of the polymer coatings can be conducted via SEM (Scanning Electron Microscope), TEM (Transmission Electron Microscope), XRD (X-ray Diffraction), XPS (X-ray Photoelectron Spectroscopy), AFM (Atomic Force Microscope), ATR-FTIR (Attenuated Total Reflectance-Fourier Transformation Infrared) and so forth.

SEM uses a narrow electron beam to bombard the surface of a sample. Due to the excitation induced by the incident beam, electrons near the sample surface can be emitted. Through the interaction between the sample and the electron beam, surface information of the sample can be obtained. The SEM mainly focuses on the sample’s surface morphology. It can be applied to polymer materials. Nevertheless, when using this microscope to test the organic material one should be careful, since energy introduced by the incident beam can vaporise the sample, leading to the generation of volatile components.

Similar to SEM, TEM utilises an accelerated electron beam to bombard the sample surface. Different to SEM, TEM collects the physical information from the back of the sample via scattered electrons. Sample-structure-related parameters can be revealed by the TEM. Additionally, morphology, sample sizes and compositions can be gathered. TEM allows the sample to be bulk material, thin film, power, fibre and nanoparticles. Polymer analysis can also be conducted.

XRD adopts X-rays to discover the crystal structure of the sample via diffraction effect and the Bragg’s Law. When the X-rays hit the sample, X-rays with unchanged frequency and amplitude will be emitted. However, the directions of the X-rays are changed. According to the Bragg’s equation, a sample with different crystal planes will result in different peaks appearing in the XRD results, namely the XRD spectrum. By interpreting the obtained XRD spectrum, crystal structure of the sample can be attained.

Moreover, XPS morphology investigation involves X-ray like the XRD. Different to XRD, XPS deploys the X-ray photons to excite the sample electrons. By utilising the photoionisation mechanism, photoelectrons with characteristic kinetic energies are excited by the photons and escape. Consequently, different elements contained in the sample are identified with their relative concentrations. XPS is generally considered as surface morphology study, due to the fact that only the photoelectrons located near the sample surface can be emitted.

AFM is a highly accurate device for studying the material surface morphology within a nanosized area. It employs a microcantilever to sweep across the designated area of a sample. Equipped with a needle tip, the microcantilever works in three modes which are the contact mode (repulsive mode), non-contact mode (using Van der Waals’ force and electrostatic force) and tapping mode. Through interaction forces (repulsive and attractive forces) between atoms from the sample and the device, sample surface images are recorded. Additionally, for electrical conductivity is not a concern for this method, material types are not restricted and can be deployed to polymers.

ATR-FTIR is one of the analytical approaches that uses an infrared spectrometer. It adopts infrared rays and total reflection theory to analyse the sample’s physical properties. Since the refractive index is constant, when the infrared rays pass from high refractive index material to a relative lower material, the refraction angle is larger than the incident angle. Consequently, once the incident angle is large to an extent, the incident rays are fully reflected. Therefore, the infrared rays are able to travel multiple times through the sample. When the infrared rays hit the sample, frequencies that are identical to sample’s chemical bonds will be absorbed, resulting in the absorption peaks displayed in the obtained spectrum. By interpreting the spectrum, the chemical structure and bonds of the polymer materials are revealed.

### 2.2. Flame Retardancy Characterisations

Generally, there are several ways to test the fire safety properties of a material. Under this subsection, typical characterization techniques namely LOI (limiting oxygen index), UL-94, Cone (Cone Calorimeter) with their key parameters will be demonstrated.

LOI is a standard testing method for the evaluation of a material’s combustibility. Generally, samples will be put into an environment containing a mixture of oxygen and nitrogen. By changing the oxygen concentration, the sample might be ignited or not by an ignition source. During the test, the oxygen concentration will be decreased accordingly, until the minimum oxygen concentration value is reached to allow the sample to burn. This value is referred to as the LOI. In this test, different temperatures can be applied to test the specimen’s property under real fire scenarios.

UL-94 is also a standard test to appraise a material’s flammability via its rating, burning rate, dripping and whether the dripping can cause the underneath cotton to burn. Depending on the type of polymers and their size, different UL-94 methods can be deployed based on their correspond standards. Furthermore, since the testing methods are not the same, the flammability rating of the polymers varies (i.e., V-0, V-1 and V-2 are for vertical specimens and HB, HF-1 and HF-2 are for horizontal foam and films).

Cone is a benchmark fire testing technique that has been adopted widely. By using the heat flux to induce ignition, Cone provides a relative large-scale fire testing which helps to reflect the specimen’s combustion behaviour under the actual fire incident. With the oxygen consumption principle, the heat release of a sample is calculated based on the oxygen depletion. Key parameters obtained to help determine the material’s fire safety properties are: HRR (heat release rate), THR (total heat release), SPR (smoke production rate), TSR (total smoke release), COPR (carbon oxide production rate), CO_2_PR (carbon dioxide production rate), TTI (time to ignition), MLR (mass loss rate), and EHC (effective heat combustion).

### 2.3. Electrical Conductivity Characterisation

To analyse the electrical conductivity of MXene based polymer coatings, the four-point contact method standard, or called four-terminal sensing (4T sensing), 4-wire sensing, is deployed. Electrical resistivity is the property of a material that measures how strongly it resists electric current. Electrical conductivity is the reciprocal of electrical resistivity. It represents a material’s ability to conduct electric current. Four-point probe with four equally spaced, co-linear electrical probes, contact with the thin coating material. It operates using DC current between the outer two probes and measuring the voltage drop between the inner two probes. The injected current (I) and voltage drop (ΔV) are used to calculate the corresponding electrical resistivity (Ω⋅m) through I/ΔV, while the conductivity (S/m) is the reciprocal of electrical resistivity.

This measurement of resistance is independent of the size of materials and makes it convenient to compare different samples. The four-point contact method is mainly used to measure electrical conductivity in ohmmeters and impedance analysers, and strain gauges and resistance thermometers. It was also used to measure sheet resistance of thin films, especially for semiconductor thin films or coatings.

## 3. MXene-Based Polymer Coatings

In recent years, MXene, a novel 2D transition metal carbide, has attracted extensive scientific interest as a coating material for multifunctional wearable device applications (i.e., wearable heaters). Apart from owning astonishing electrical conductivity and thermal conversion efficiency, the 2D nanostructure with metallic and ceramic features also endows MXene with a significant fire-retardant property. Therefore, different traditional coating methods have been adopted to utilise MXene as a lightweight homogeneous coating for wearable devices due to its versatile electrical and thermal properties, such as spray coating [30,31], dip coating [32,33] and solution-casting [34]. Moreover, other new developed coating technologies should be addressed.

Spray coating generally involves using a spray gun to deposit desired materials to the surface. Depending on the speed, flow, cycles of the spray and the coating concentration, the coating parameters can vary. Spray coating is beneficial for large-scale processing. Additionally, spray coating can help provide uniform and ultrathin coatings, which is a good choice for fabricating monolayer and multilayer thin films.

In the case of preparing ultrathin films with low concentrations of coating materials, dip coating can be a better solution compared to spray coating. It is straightforward to understand that dip coating is a simple approach that lets the substrate submerge into the coating solution and generate the desired coatings. By modifying the substrate surface, dipping time and the coating solution concentration, dip coating can tune the thickness and roughness of the coatings.

Solution casting is a coating method that immerse the substrate into a tank filled with coating solutions. By utilising the thermal and frictional properties of the coating material, desired materials can be coated on the surface of the substrate with the shape of the mould. Solution casting allows the coating of different materials and the inclusion of different parts. It is a cost-effective coating method and simple to apply.

Additionally, before applying the coating, the fabrics and textiles were often treated with deionised water, alkali solution, and plasma-surface techniques to enhance the purity, surface activity, and hydrophilicity of the pristine substrates. For other coating techniques, they will be discussed in the later sections.

### 3.1. Single Coating of Unmodified MXene

**Figure 1:**
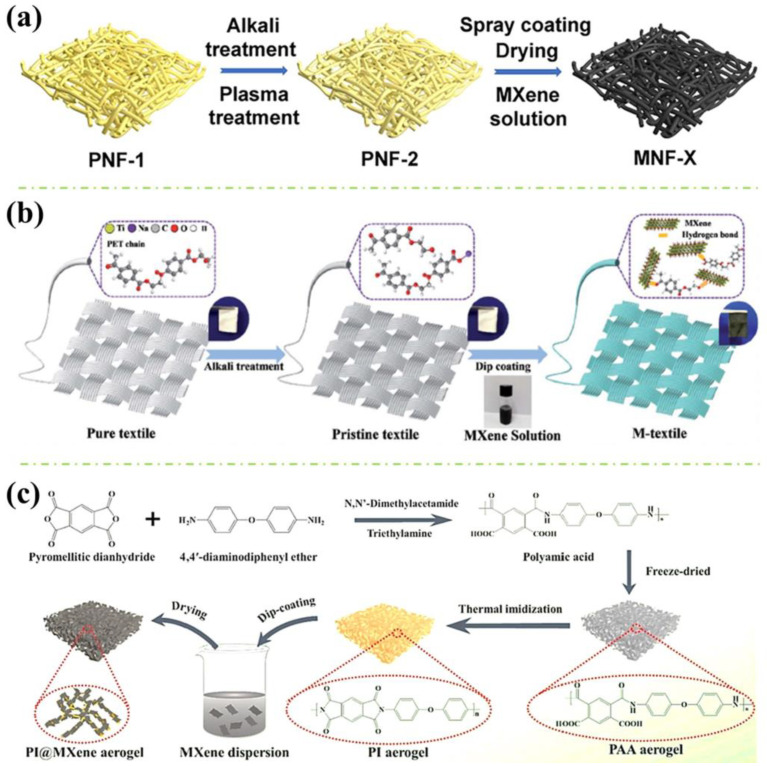
(**a**) The preparation process for MXene-coated nonwoven fabric [30]; (**b**) The dip coating process of MXene nanosheet coating on PET textile [32]; and (**c**) The synthesis route of the PI@MXene aerogel [33].

**Figure 2:**
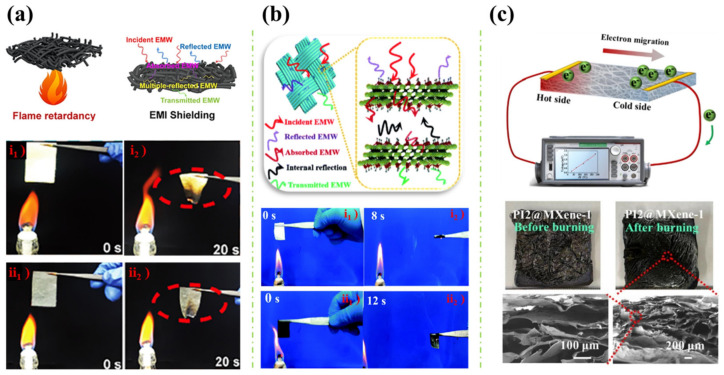
(**a**) Schematic diagram of flame retardancy (up left) and electromagnetic waves absorption (up right) of the MXene-coated nonwoven fabric and fire testing of uncoated fabric (i1 and i2) and MXene-coated nonwoven fabric (ii1 and ii2) [30]; (**b**) The electromagnetic waves shielding mechanism and the vertical burning tests of pure PET textile (i1 and i2) and MXene nanosheet coated PET textile (ii1 and ii2) [32]; and (**c**) The temperature-sensing experiment and the morphological investigation of PI@MXene aerogel before and after burning [33].

Recently, Wang et al. [30] fabricated a lightweight MXene-coated nonwoven fabric with significant flame resistance, electromagnetic interference (EMI) shielding and electrothermal/photothermal conversion properties. The surface of the nonwoven fabric is modified through the spraying–drying method, as illustrated in Figure 1a. The pristine fabric (PNF1) was first cleaned with an alkali solution (i.e., NaOH) and treated with plasma-surface-technology treatment. This could improve the surface activity of the Aramid nonwoven fabric, achieving increased wetting properties and large amounts of hydrophilic groups. Subsequently, MXene solution was evenly layered on the surface of the pretreated fabrics (PNF2) via a small sprayer and dried by a hair dryer. The spraying–drying process was repeated several times on both sides of the fabrics, the MXene-modified nonwovens with 7.97, 10.14, and 18.87 wt.% of MXene were then fabricated by manipulating the number of spraying times. Herein, the spray coating method has layered the MXene nanosheets on the fibres’ surface with porous structures. The morphological results indicated disorderly arranged fibres in the nonwoven fabric, forming surface pores with different dimensions, where increments in MXene content led to a rougher and more wrinkled fibre surface due to the piling of the MXene sheets on the surfaces. Excellent adhesion was achieved between MXene and the fabrics via the hydrogen bond interaction. According to the literature, the as-prepared fabric exhibited astounding flame retardancy and EMI shielding properties. As illustrated in Figure 2a, the coated fabric has performed a reduction in combustion duration and degree of carbonisation in a reduced-scale vertical burning experiment. The enhancement of the flame-retardant performance of the MXene-coated fibre was suggested to be the physical barrier effect and carbonisation effect exhibited from the MXene nanosheets, where MXene is able to oxidise to TiO_2_ in a hyperthermal environment, leading to an intumescent and dense TiO_2_ layer formation on the fabric surface. The formed char layer acts as a physical barrier that disrupts heat transfer over the fabric surface [35,36]. On the other hand, an EMI shielding efficiency of 35.7 dB for single-layer fabric was achieved, this was attributed to the interlacing conductive grids constructed between the stacked MXene sheets and the nonwoven fabric, which provides significant abilities in reflecting and absorbing electromagnetic waves, as well as thermal energy.

While earlier applications of MXene nanosheets on wearable heaters can be found in Liu et al. [32] work in 2020, where MXene-decorated polymeric textiles were synthesised via a dip coating method revealed in Figure 1b. Similar to Figure 1a, alkali pretreatment using NaOH solution was applied on pure polyethylene terephthalate (PET) textiles to fracture ester groups within the PET structure, this could allow more oxygen-containing groups on the textile surface (identified by morphological study), which is advantageous to the formation of hydrogen bonds between the textile and MXene, resulting in a strong MXene-PET interaction through the synergistic effect of hydrogen bonds and physical rivet phenomena. The pristine PET textiles were then dipped into MXene solution and then dried at 80 °C in a vacuum oven. The content percentage of MXene within the polymeric textiles was regulated by controlling the repeating times of the dipping process, ranging from 1 to 17.3 wt.%. In the morphological investigation, the dip-coated textiles have featured a smoother surface due to the adhered MXene nanosheets, which are riveted tightly in the cavities on the textile surface, compared to the rough surface with cavities exhibited by the pristine PET fabrics. The result of energy dispersive spectrometer (EDS) mapping has presented a uniform arrangement of carbon element (C) and titanium element (Ti) on the fibre surface, which further indicates a well distribution of MXene nanosheets on the PET fabrics. Significantly enhanced fire-resistance performance and EMI shielding effects were achieved. As depicted in Figure 2b, the pristine and MXene-coated textiles possess different burning behaviours during the examination of an alcohol burner. After both textiles were ignited on the burner for 1 s, the pristine PET textile performed violent combustion behaviour and totally burnt out the sample within 8 s, where a heavy melt drip was also observed. For the MXene-coated textile, the combustion completely ceased within 12 s with no melt dripping and preserved the textile integrity. The outstanding flame retardancy of the coating textile can be attributed to the oxidised TiO_2_ char, which inhibits flame spread and melting droplets, offering the enhanced anti-dripping ability of PET textiles. Moreover, a high EMI shielding efficiency of 42.1 dB in the X-band was also reported in the literature, this is attributed to the high electrical conductivity of MXene nanosheets, which aids the reflection of electromagnetic waves on the textile surface. After the waves penetrated the surface, the trapped electromagnetic waves were absorbed in the form of heat energy within the nanosheets and dissipated by the multiple reflections due to the existence of MXene nanosheets with a large surface area and interface area.

On the other hand, Jiang et al. [33] prepared polyimide (PI)/MXene composite aerogel with delicate temperature-sensing and flame-resistance capability. Figure 1c presented the schematic of the fabrication process, before obtaining PI, poly (amic acid) (PAA) was synthesised by adding pyromellitic dianhydride (PMDA) into 4, 4′-diaminodiphenyl ether (ODA) solution, the PAA solution was then transformed to aerogel via freeze-drying. Later, the PAA aerogel was thermally imidized under nitrogen flow in a muffle furnace, the PI aerogel was obtained. The PI aerogel was then plasma-treated via vacuum plasma surface treatment prior to the dip-coating process. Micromorphologically, a large number of polar groups were generated on the inner pore wall of PI aerogel after plasma treatment, which was beneficial to the diffusion of MXene dispersion in the aerogel and enhanced the adhesion strength between the MXene and the PI skeleton. Subsequently, the activated PI aerogel was dipped into the MXene solution. After the dip-coating procedures were repeated several times, the composite was then positioned vertically in the oven. Throughout the morphological characterisation, smaller sizes and more uniform distribution of pores were identified on the surface of MXene-coated PI aerogel, with a thicker inner pore wall compared to pristine PI aerogel, which further indicated the successful coating of MXene on the surface of the aerogel. Furthermore, it was found that the as-fabricated PI/MXene composite aerogel possesses advanced flame retardancy, thermostability and temperature-sensing performances. As revealed in Figure 2c, PI/MXene exhibited extraordinary structural stability during combustion, while the PI aerogel was softened and deformed. The great flame retardancy was ascribed to the lamellar barrier and skeleton-supporting effect of the MXene nanosheets, the PI/MXene presented larger inter-lamellar pores and flaky pore walls, which effectively disrupted heat transfer and protected the inner aerogel from combustion.

### 3.2. Single Type Coating of Modified MXene

Besides the extraordinary fire resistance and electrical properties offered through homogenous MXene dispersion coatings [30,31,32,33,34], MXene is also able to be integrated into other hybrid material systems, aiming to overcome specific applicational challenges (i.e., next-generation lithium battery, organic thermoelectric systems). This can be achieved by constructing multi-synergistic interactions between MXene nanosheets and other layered materials via different assembling methods, such as facile vacuum filtration approach [37], dip coating [38] and soaking–drying–reduction method [39].

**Figure 3:**
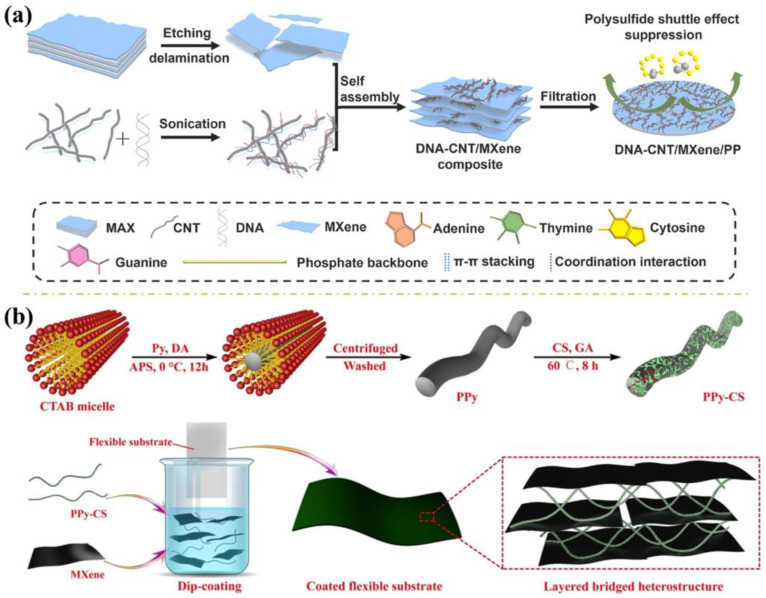
(**a**) The fabrication process for the DNA-CNT/MXene/PP separator via facile vacuum filtration approach [37] and (**b**) The co-assembly process of the PPy-CS/MXene nanocoating on the substrate with layered bridged heterostructure [38].

For instance, Li et al. [37] synthesised a hybrid-modified polypropylene (PP) with deoxyribonucleic acid (DNA)-carbon, nanotube (CNT) and MXene via facile vacuum filtration. As demonstrated in Figure 3a, the MXene nanosheets and DNA-CNT solution were mixed and sonicated in ethanol, then filtered onto a PP separator via a vacuum-assisted filtration device, where DNA-CNT and MXene nanocomposites were self-assembled by forming multiple interactions between the DNA molecules and MXene nanosheets. Morphologically, the DNA considerably increased the interlayer spacing of MXene nanosheets and provided considerable channels among the MXene nanosheets to enable fast lithium-ion transport and immobilising the soluble polysulfide for lithium–sulphur (Li-S) batteries.

Moreover, Xie et al. [38] fabricated a flexible organic thermoelectric nanocoating via co-assembling cellulose-modified polypyrrole (PPy-CS) and MXene nanosheets with layered bridged heterostructure, achieving high thermoelectric efficiency and flame retardancy (see Figure 3b). PPy-CS/MXene solution was prepared for the plasma-treated substrates to perform the dip coating method. Owing to the gravity and hydrogen bonding, MXene nanosheets were arranged horizontally and assembled with the PPy-CS during the drying of the wet coating, developing PPy-CS/MXene nanocoating with ordered layered bridged heterostructure. The hybrid nano coating has offered excellent fire retardancy to PET films and polyurethane (PU) foams, achieving self-extinguished immediately after 10 s of ignition in the vertical burning test and increasing the limiting oxygen index (LOI) to 31.4%, with the highest residue of 96.4 wt.%. The outstanding flame resistance performance can be attributed to the carbonisation products of PPy-CS bonded with MXene nanosheets, where pyrolysed gases were trapped and accumulated beneath the nanocoating, thus the layered porous structure was formed. PPy-CS/MXene nanocoating rapidly experienced carbonisation and crosslinking reactions when encountering fire, generating thermally stable structures such as cyclic compounds and bonding MXene nanosheets. This layered porous structure has effectively excluded the external oxygen and heat energy from the interior nanocoating and the substrates, further promoting the heat insulation effect.

### 3.3. Multilayer Coatings of Unmodified MXene Overlapping with Other Materials

Previous sections summarise the techniques for coating a single type of modified/unmodified MXene to the substrate for the improvement of flame retardancy and electrical conductivity performances. Moreover, relevant mechanisms for featuring MXene with fire safety and conductive properties have been illustrated comprehensively. As mentioned before, for the mono- or multi-repeated layer coatings of the pure and/or treated MXenes, methodologies for applying the prepared functional materials to the substrates include spinning, spraying, dipping, blade coating and vacuum-assisted filtration. In general, these procedures are easy and convenient for fabricating single-component polymer coatings. Nevertheless, the interaction strategies for bonding different functional layers should be considered when introducing multilayer coatings to the matrix. This section reviews the studies that coat pure MXene and other components as different coating layers. The addition of MXene mainly focuses on improving the fire resistance and electrical conductivity properties of the substrates. Moreover, the other functional layers may work collaboratively with MXene to enhance the same properties or render the polymers with additional performances such as durability.

Frankly speaking, for the simplicity of manufacturing, if only one or two additional coating layers are required, directly utilising of the abovementioned coating techniques is enough as the concerns about the layers’ crosslinking may not be extremely necessary. For example, Wei et al. [40] sprayed the MXene on the surface of the pine wood. In order to improve environmental stability, a waterborne acrylic resin was brushed on the outside of the MXene layer as another functional coating. Moreover, for the enhancement of the as-prepared composite’s stability and hydrophobicity, another material named methyltrimethoxysilane (MTMS) was also studied by Jia et al. via the vacuum filtration coating of MXene followed by the MTMS [41]. Additionally, dipping and spinning techniques were performed to improve the compatibility of the coated composites as well as strengthen the MXene’s effectiveness in endowing the polymers with flame retardant and electrical conductivity properties [42,43,44,45].

**Figure 4:**
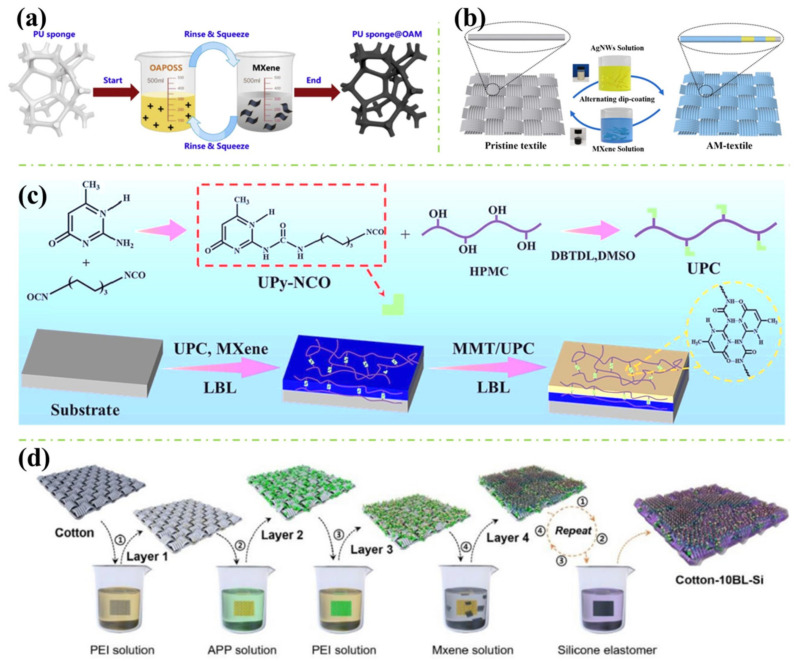
(**a**) The schematic diagram of fabricating MXene and OAPOSS coated PU sponge via LbL assembly [46]; (**b**) The preparation route of AgNWs and MXene-coated PET textile [47]; (**c**) The fabrication procedures of UPC, UPC/MMT and UPC/MXene coated flammable substrate [48]; and (**d**) The synthesis route of LbL-treated multifunctional cotton fabric with APP, PEI, MXene and PDMS solutions [49].

Generally, for employing multiple coating layers to the polymers, there is a pervasive method named layer-by-layer (LbL) assembly. The basic approach of the LbL assembly is utilising attractive forces such as Van der Waals force, electrostatic force and/or hydrogen bonding to attach two or several desired materials on the surface of the substrate, one layer after another. Figure 4a shows a simple procedure of coating the polyurethane (PU) sponge with octaammonium-polyhedral oligomeric silsesquioxane (OAPOSS) and MXene (Ti_3_C_2_ nanosheets) via the LbL assembly [46]. The OAPOSS was synthesised from 3-(aminopropyl)triethoxysilane (APTES) for the introduction of SiO_2_ during the combustion, which helped reinforce the flame retardancy endowed by MXene. Additionally, Liu et al. fabricated a silver nanowire (AgNWs) and MXene-decorated PET textile through the LbL technique (see Figure 4b) [47]. With the treatment of the AgNWs, polyvinylpyrrolidone (PVP) with carbonyl groups is attached to the surface of the AgNWs, which improved the compatibility with both PET through its functional groups and MXene layers through MXene’s termination groups via hydrogen bonding. In addition, a complicated procedure of coating MXene with 2-ureido-4[1H]-pyrimidinone-containing cellulose (UPC) and montmorillonite (MMT) with UPC through two passes of LbL assembly on the flammable materials (pinus sylvestris wood, cotton fabric and PU) was conducted by Zeng et al. (see Figure 4c) [48]. According to the researchers, due to the existence of ureido-pyrimidinone (UPy) groups within the UPC, this enabled hydrogen bonding with the MXene and MMT through their oxygen-contained groups, and with UPC itself, which also embraced the pinus sylvestris wood with remarkable self-healing property. Moreover, holding a similar concept of adopting three different coating materials to the substrate, Liu et al. applied polyethyleneimine (PEI)/ammonium polyphosphate (APP)/PEI/MXene as a quad-layer (four-layer coatings as a group) coating to the cotton fabric for the introducing of flame retardancy and electrically conductive performances (see Figure 4d) [49]. A silicone-containing polydimethylsiloxane (PDMS) was then finally coated on the modified cotton fabric to render the polymer with a superhydrophobic finishing surface. Furthermore, apart from the investigations explained before, other studies that utilised the LbL assembly technique for introducing multilayer coatings on the surface of the polymers can be the alternative coatings of carboxymethyl chitosan (CCS) with MXene [50], polyethyleneimine with MXene [51] through dip coating. In addition, the magnetic Fe_3_O_4_ nanoparticles were also incorporated with MXene through spray coating, which was finished with a hydrophobic PDMS coating layer [52]. Based on the studies reported so far, for employing multiple coating layers to improve the polymers for better fire resistance and electrically conductive properties, LbL assembly can be a commonly used technique, while other treatments to the functional layer materials might be included to improve the layers’ adhesion capabilities and further rendering the polymers with additional properties.

**Figure 5:**
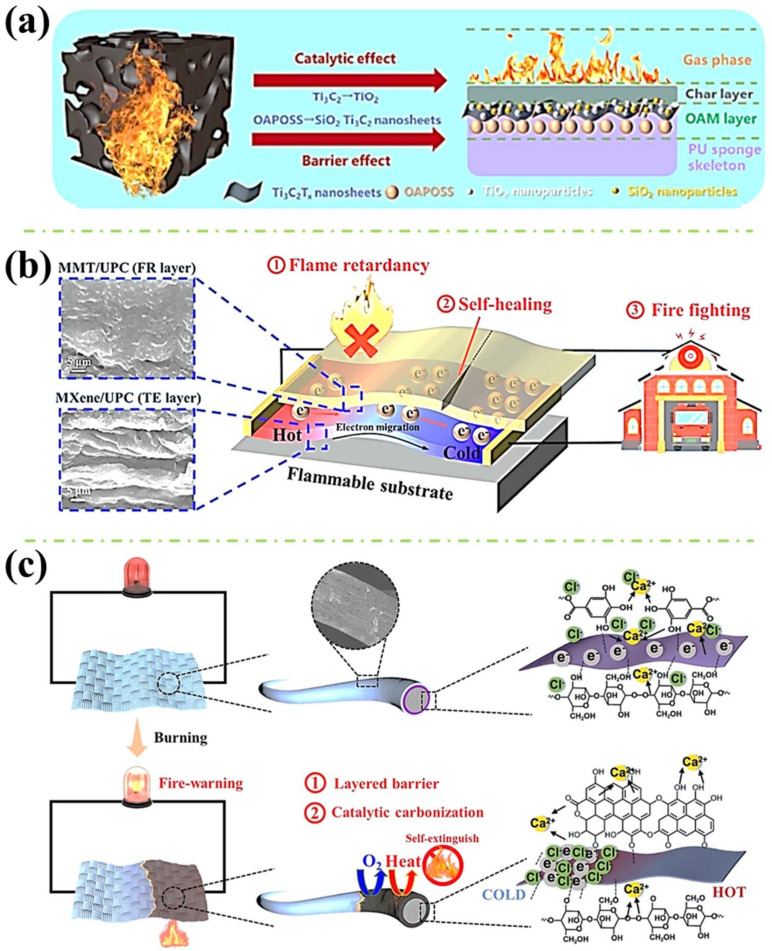
(**a**) The flame-retardant mechanism of MXene and OAPOSS coated PU sponge; (**b**) The flame-retardant strategy of UPC/MMT and UPC/MXene coated flammable substrate; and (**c**) The fire-resistance mechanism of MXene and TA/CaCl_2_-coated cotton fabric.

As previously demonstrated, research works included in this session also mainly deployed the physical barrier effect of the MXene to import the substrates with fire safety performances. Nevertheless, with the combination of other treated/untreated materials, additional flame-retardant mechanisms induced by these combining materials are also involved and clarified. These additional fire-resistance mechanisms may also work in both condense and gas phases during the combustion, even generating synergistic effects with MXene, which consequently impart the flame retardancy of the polymers. For instance, as stated in Figure 5a, Lu et al. applied OAPOSS with MXene on the PU sponge via LbL assembly [46]. With the silicone element contained in the introduced OAPOSS, during the fire scenario, OAPOSS can decompose into the SiO_2_ to deliver the barrier effect. Additionally, the resulted SiO_2_ can form a hierarchical structure of SiO_2_-TiO_2_-Ti_3_C_2_T_x_ with the oxidised and unburnt MXene, which further enhances the charring performance in the condensed phase and magnifies the physical barrier effect, resulting in the reduced combustible volatiles generation in the gas phase. In addition, according to Zeng et al., with the UPC/MXene and UPC/MMT LbL coating, the fabricated pinus sylvestris wood possessed excellent flame-retardant capability (see Figure 5b) [48]. This can be attributed to (1) Carbonisation catalysis of the incorporated MMT and MXene on the UPC, which resulted in a condensed char above the substrate; and (2) Barrier effect provided by the MMT, MXene and the produced char, which restrained the heat and gas exchange. Furthermore, in 2023, Zeng et al. mixed tannic acid (TA) with calcium chloride (CaCl_2_) to form one part of the coating component and applied it to the surface of the cotton fabric with MXene via LbL technique (see Figure 5c) [53]. It was found that, during the combustion: (1) MXene can participate in the charring behaviour via the Ti-O-C; (2) TiO_2_ generated by the MXene can promote the decarboxylation and carbonisation of TA; (3) The dehydrated TA can crosslink with the cotton fabric, which forms a compact char on the fabric’s surface; (4) Ca^2+^ can also react with the TA, MXene and cotton fabric, which also enhances the char quality; (5) Excellent and integrate char was formed, which showed remarkable barrier effect; (6) Cl^−^ from the CaCl_2_ contributed to the radical capture performance in the gas phase, which postpones the degradation of the cotton fabric. From the mechanisms mentioned above, it can be seen that the primary strategies used in polymer coating techniques to endow flame-retardant performances are the char promotion and the physical barrier effect in the condensed phase with their accompanying effect of gas inhibition in the gas phase. Other theories introduced by additional coating materials can be free radical capturing in the gas phase and char quality improvement (crosslinking the char or dehydration catalysis) in the condensed phase.

**Figure 6:**
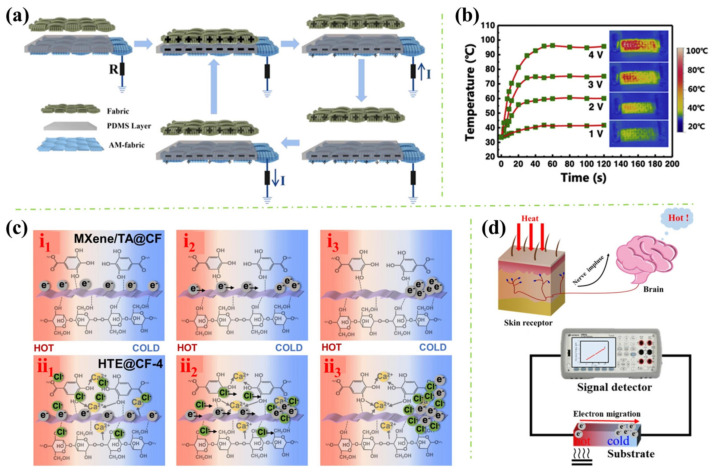
(**a**) The schematic TENG working flow of AgNWs/MXene coated PET textile [47]; (**b**) The Joule-heating performance of MXene/MTMS coated wood-pulp fabric [41]; (**c**) The thermoelectric mechanism of MXene and TA/CaCl_2_-coated cotton fabric [53]; and (**d**) The mechanism of skin-inspired UPC/MXene and UPC/MMT-coated pinus sylvestris wood temperature sensor [48].

In addition, apart from the flame-retardant mechanism, the excellent electrical conductivity mechanism endowed to the polymers and its feasible applications should also be addressed. For example, Liu et al. explored the triboelectric nanogenerator (TENG) principle of the AgNW/MXene-coated PET textile [47]. In Figure 6a, the authors used a common textile to touch the surface of the modified textile (AM-fabric) which is connected to the ground. It can be known that when the common textile (green) contacted the surface of the AM-fabric, positive charge was formed on the surface of the textile while negative charge remained on the PDMS layer of the AM-fabric. At this stage, due to the textile being in contact with the AM-fabric, the charges maintain equilibrium. Then, after the textile is removed, the negatively charged PDMS can cause the underlying AM-fabric to become positively charged, leading to the free electrons moving to the ground and triggering an electrical signal. Additionally, if the textile has been located far enough from the PDMS layer, the charges are shielded, and the AM-fabric becomes uncharged. However, once the positively charged textile is put back, an electrical signal will be generated again in an inversed direction. Moreover, Jia et al. evaluated the Joule-heating performance and the mechanism of their manufactured MXene/MTMS-coated fabric (see Figure 6b) [41]. According to the authors, due to the high electrical conductivity of the MXene, the electrical resistance of the treated fabric is very small. Therefore, the low-voltage-driven heating phenomenon was received as a consequence of the electron–phonon interaction (EPI). This process occurs when an electron pulls a phonon from a low-temperature area to a high-temperature area, where there is a temperature difference present. Thus, if the voltage is higher, the electrons will become more vigorous, causing the phonons to be effectively excited, which results in a quick temperature increase. Furthermore, Zeng et al. examined the thermoelectric property of the fabricated cotton fabric (see Figure 6c) [53]. Generally, the mechanism of MXene-endowed thermoelectric performance can be explained by MXene’s electron-type carriers that move from the high-temperature region to the low-temperature region, thus generating a voltage (see Figure 6c(i_1_–i_3_)). However, when introducing the CaCl_2_/TA coating to the cotton fabric, the Seebeck coefficient was significantly increased. This can be elucidated by another thermoelectric mechanism, which is due to the import and increase of Cl^-^ that migrates together with the electrons (see Figure 6c(ii_1_–ii_3_)). Furthermore, by the inspiration of the skin, Zeng et al. prepared a multifunctional coating for rendering the polymer with fire-warning properties (see Figure 6d) [48]. Although the construction of the coating is rather complex, consisting of LbL-coated UPC/MXene and LbL-coated UPC/MMT, the idea behind the design is quite interesting. To be specific, the UPC/MMT layer functions like the epidermis of the skin which protects the underlying tissue. Additionally, the UPC/MXene layer can be considered as the dermis layer of the skin, which senses the temperature and alerts the associated danger. Also, good sensing performance was received from the as-fabricated polymers. In summary, from the key findings depicted above, it can be found that with the electrical conductivity property rendered by the MXene as well as other coating materials, the applications of the fabricated polymers have been extended but not limited to the TENG, fire-warning sensor, EMI shielding, heat-generating device, etc.

### 3.4. Multilayer Coatings of Modified MXene Overlapping with Other Materials

The previous sections discussed applications of single-type coating of pure MXene and modified MXene towards the enhancement of flame retardancy and electromagnetics. This section will summarise the research work about multilayer coatings of modified MXene combined with other functional materials. As aforementioned, MXene performs as a representative candidate material for high-efficiency electromagnetic absorbers, including strong conduction loss and polarisation loss, large specific surface area and high aspect ratio, etc., they could be recognised as an ideal platform carrier to couple with other components to improve the composites’ properties.

**Figure 7:**
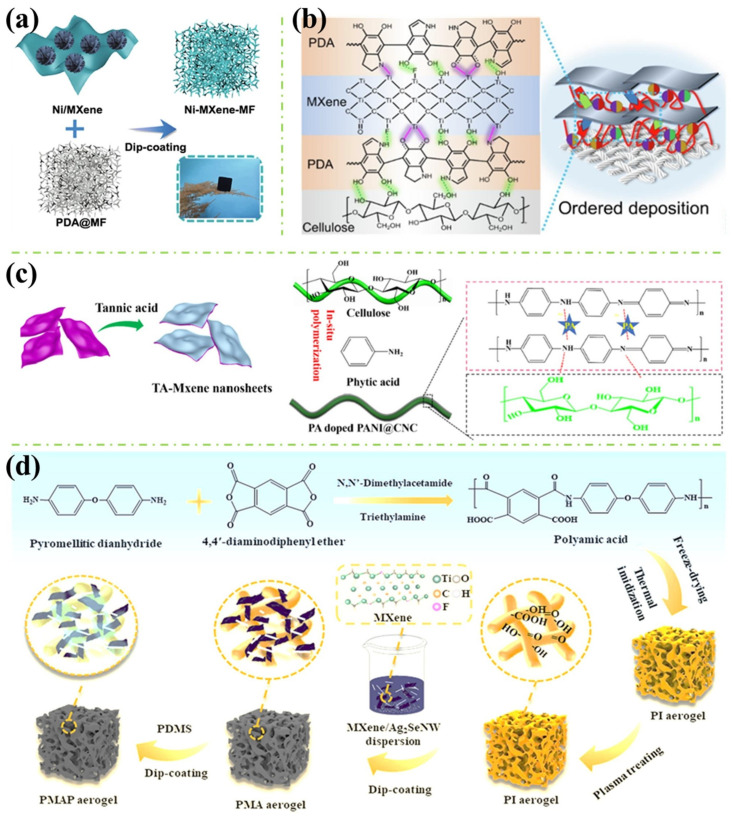
(**a**) The preparation process for Ni/MXene-MF [54]; (**b**) MXene@PDA hybridisation structure [55]; (**c**) TA-MXene nanosheets and the synthesis of PA@PANI@CNC [56]; and (**d**) The fabrication of the PMA aerogels and PMAP aerogels [57].

**Figure 8:**
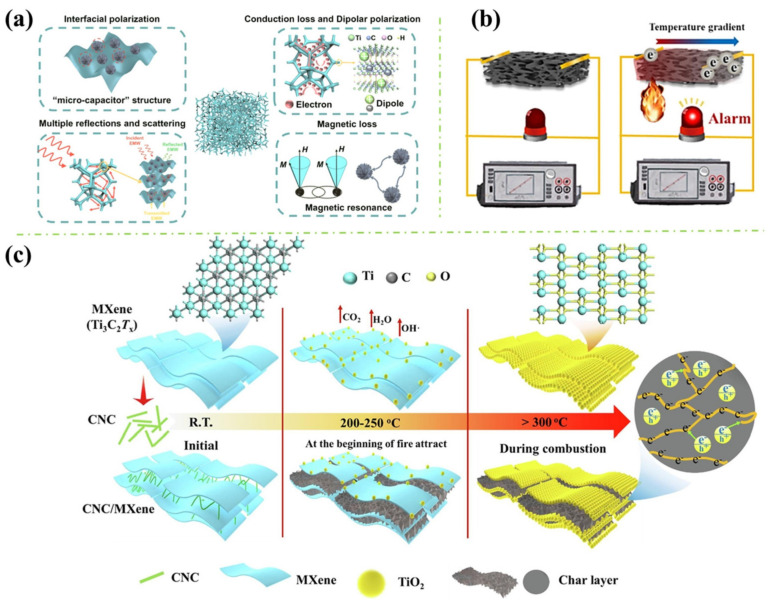
(**a**) The microwave absorption mechanism of Ni/MXene-MF [54]; (**b**) Schematic for the temperature sensing of the PMA aerogels [57]; and (**c**) Schematic illustration for fire warning of CNC/MXene coating [58].

Cheng et al. [54] assembled the magnetised Ni flower/MXene hybrids on the surface of melamine foam (MF) by electrostatic self-assembly and dip-coating adsorption process, as shown in Figure 7a. The MXene flakes with negative zeta potential anchored to the positively charged Ni flower and the Ni/MXene hybrids were assembled on the surface of MF through the capillary force of MF and the adhesive power of polydopamine (PDA) by the dip-coating process. The 3D porous framework of Ni/MXene-MF, proposed by authors, formed an interconnected conductive network, shown in Figure 8a. The porous structure helped the migration of electrons, resulting in conduction losses and attenuating microwave energy, and a large number of interfaces generated the interface polarisation effects. Moreover, the porous structure increased the propagation path of microwaves, and the Ni flower attenuated EM waves through natural resonance and exchange resonance. The minimum reflection loss of −62.7 dB with a corresponding effective absorption bandwidth (EAB) of 6.24 GHz at 2 mm and an EAB of 6.88 GHz at 1.8 mm were achieved.

Yan et al. [55] added dopamine (DA) hydrochloride solution into MXene nanosheet dispersions to obtain the MXene@PDA hybrids, where PDA was used as a binder and crosslinking agent to expand the size of MXene lamellae, resulting in a directional ordered self-stacking structure, as shown in Figure 7b. Yan et al. analysed the binding form for constructing order-stable MXene nanosheet layer structures on the surface of cotton fabrics, which was attributed to the Si-C bond in the hydrophobic agent, proving that the hydrophobic layer was successfully introduced to the fabric surface. The fabric’s conductivity was gradually increased by introducing the conductive medium MXene. Furthermore, the stability of the conductivity of materials was enhanced due to the positive effect of PDA on the fastness of the functional coating formed by MXene on the fabric surface.

**Figure 9:**
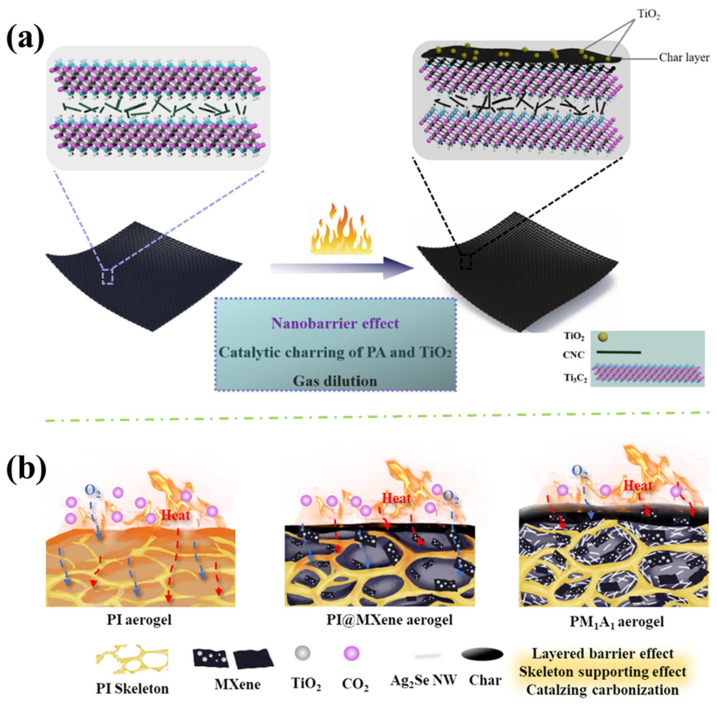
(**a**) Flame-retardant mechanism of coated cotton [56] and (**b**) flame-retardant mechanism of the PMA aerogel [57].

In addition, MXene can be surface-engineered by TA as shown in Figure 7c. Mao et al. [56] added Ti_3_AlC_2_ powder into the mixture of LiF and HCl, and after etching of Al, TA aqueous solution was mixed with Ti_3_C_2_T_x_ dispersions. The TA not only acted as the antioxidant and stabiliser of Ti_3_C_2_T_x_ nanosheets, but also provided abundant phenolic hydroxyl groups to form hydrogen-bonding interaction with oxygen-containing groups in cellulose nanocrystals (CNC). The PA@PANI@CNC nanocomposite was synthesised through in-situ polymerisation of aniline using CNC as the bio template and PA as the doped acid. The PA can improve the flame retardance of the matrix and deliver six strong dissociated protons, which increases the electrical conductivity of PANI. They investigated the flame-retardant mechanism of Ti_3_C_2_T_x_/PA@PANI@CNC nanocoating on cotton. As shown in Figure 9a, Ti_3_C_2_T_x_ nanosheets and 1D (one-dimensional) CNC suppress the heat and mass exchange by generating anatase TiO_2_, which can reduce the O_2_ concentration and the penetration of oxygen into the fabrics. Meanwhile, with the formation of the protective char layer, further isolation of heat and mass transfer is achieved.

Compared to most modified MXene methods, Qu et al. [59] annealed the MXene first. They then fabricated a unique gradient heterogeneous layers structure electromagnetic shielding composites via coating annealed MXene into the AgNWs networks. The results showed that AgNWs orthogonal network structure could effectively reduce the interfacial thermal resistance between filler particles, improve the phonon transfer rate, and the composites could retain their original shape after combusting for 60 s without any shrinkage or ignition. Due to the formation of TiO_2_, a dense heat insulation layer wrapped on the material’s surface and effectively inhibited heat transfer, improving the flame retardancy of the composites.

Li et al. [60] fabricated fire-warning papers by immersing cellulose paper in phytic acid (PA) solution and dip-coating it with graphene oxide (GO)/MXene suspension. The electrical resistance of the fire-warning papers sharply decreases when exposed to heat. The results showed that the time for electrical resistance decrease exhibits further decrease with more loading of MXene content, and higher test temperature can lead to a faster electrical resistance change.

Zhao et al. [57] fabricated PI/MXene/Ag_2_Se nanowires (PMA) composite aerogel by immersing PI aerogel into MXene/Ag_2_Se nanowires dispersion and oven drying, shown in Figure 7d The PDMS-coated PMA (PMAP) was fabricated via dip-coating and oven drying. Real-time temperature monitoring performance was identified and shown in Figure 8b. When the aerogel is exposed to fire, electrons will transfer from the hot side to the cold side, and the alarm device will be triggered. Also, this fire-warning performance was improved due to the synergistic effect between the thermoelectric efficiency of the Ag_2_Se NW and MXene. The flame-retardant mechanism of the as-prepared PMA aerogel was discussed and shown in Figure 9b. TiO_2_ generated from MXene further catalysed the carbonisation of PI, forming a dense protective layer on the combustion surface. At the same time, the layered barrier of MXene and the skeleton-supporting effect of Ag_2_Se nanowires prevent the collapse of the formed char layer during the combustion process by the oxygen and combustible gas. In addition, the stable char layer reduced the HRR and gas release amount during combustion. Therefore, the flame retardancy and thermal stability of the PMA were improved synergistically.

Zhang et al. [58] added MXene (Ti_3_C_2_T_x_) sheets into CNC suspension. Consequently, they fabricated a smart wood through LbL assembly of PDA and APP polyelectrolyte solution. PDA is introduced as an intermediate adhesive bridge with hydrogen bonds, van der Waals forces, and mechanical interlocking interactions to facilitate stable bonds of the MXene-based fire warning coating on a wood surface. They evaluated the thermal trigged electrical resistance performance of MXene and CNC/MXene coated wood. As shown in Figure 8c, when encountering a fire attack, Ti_3_C_2_T_x_ nanosheets undergo edge thermal oxidation in the presence of sufficient oxygen and heat, progressively transforming them into the hybrid crystals of MXene sheet and anatase TiO_2_ phases at the temperatures of 200~250 °C during the heating process. Further, the carbonisation products of CNC bond the MXene nanosheets, forming stable multiple conductive paths. With the temperature increasing over 300 °C, the total oxidated TiO_2_ char formatted, which promotes the dramatic resistance transition from the insulating state to the conductive state, triggering the superior fire warning capacity. Additionally, fire safety tests revealed that smart wood with MXene-based coating possesses superior flame retardancy, including LOI of 47.4%, UL-94 V0 rate, av-HRR of 54.70 kW/m^2^, as well as excellent smoke suppression performance (TSR of 119.9 m^2^/m^2^, peak CO_2_ and CO production of 0.251 g/s, 0.0037 g/s).

The synthesis and preparation methods of the reviewed modified MXene are summarised in Table 1, including the applied materials and strategies for the modified MXene and the other materials used in the research work. Additionally, a selective summary of the fire safety properties and electrical conductivities of studies in this review is presented in Table 2.

## 4. Conclusions and Perspectives

In summary, the recent progress of MXene-based flame retardants and electrically conductive polymer coatings are comprehensively reviewed. Obviously, this area is growing rapidly and attracting an increasing number of interested researchers, especially over the past three years. MXene nanosheets have proved to play a critical role in preparing advanced polymer coatings with excellent fire safety and outstanding electrical conductivity, attributed to their structural advantages and intrinsic features.

Consequently, pointing out the merits and drawbacks of the MXenes is crucial for their future development. Specifically, in terms of fire safety, the 2D nanosheet structure allows MXene to act as a physical barrier that can effectively delay the efficient transfer of heat, oxygen, and flammable volatiles. Moreover, compact and thermally stable char layers are generated during combustion induced by the 2D structure, which may further effectively reduce the HRR, THR and smoke production, resulting in better fire safety performance. As to electrical conductivity property, owing to the soft nanosheet character, conductive MXene coatings, membranes, films, skeletons, and networks can be easily fabricated through various methodologies along with targeted polymers.

In terms of MXene without modification, it is usually used as an important component in mono- and multilayer coatings. Due to the hydrophilic feature, pretreatments such as plasma technique, alkali treatment, and hydrogen bond interfaces are necessary for the surface finishing of a substrate before coating MXene. Generally, traditional methods such as dipping, casting, and LbL self-assembly are the main fabricating tools for synthesising MXene-based flame retardant and electrically conductive coatings. Furthermore, combining MXenes with functional additives can achieve advanced polymer coatings with great flame retardancy and electrical sensitivity since MXene has excellent compatibility depending on the hydrophilic property.

Nonetheless, hydrophilic MXene still requires further modification to meet specific requirements. MXene nanosheets can be easily oxidised, which may deteriorate their intrinsic properties and electrical conductivity. Hence, most modification processes are carried out under relatively low temperatures and within an aqueous solution. Additionally, due to the excellent hydrophilic advantage and rich hydrogen bond structure, MXene may be modified by inorganic materials, organic polymers and molecules, and natural biomass chains to form stable MXene-based multifunctional coatings. Therefore, the incorporated inorganic materials, organic polymer molecules, and biomass will also play key roles in achieving targeted functions such as flame retardancy, electrical conductivity, EMI shielding, etc. Usually, these modification methods, such as exfoliation using molecules and long polymer chains or modification using inorganics, will damage the terminations (-O, -OH, -Cl, -F), which play critical roles in determining the electrical properties.

Based on the abovementioned advantages of the MXene, MXene can be used as components for supercapacitors and batteries for energy storage and conversion purposes. Additionally, it can be applied as sensors for electronics devices in various areas and catalysts for applications such as HER (hydrogen evolution reactions) and ORR (oxygen reduction reactions). Moreover, due to its excellent thermal properties and intrinsic 2D structures, it can be a superior flame retardant and useful in applications requiring thermal management. Furthermore, with its high adsorption capacity and biological features, it can be adopted for pollutant scavenging in environment remediation field and bacterial elimination in biomedical applications.

Currently, MXene is still facing significant challenges in terms of synthesis methods and costs. It is well-known that MXene is mainly kept in an aqueous solution and cannot be easily applied in hydrophobic polymers, which is an urgent problem that needs to be addressed. Moreover, methods to successfully protect MXene from oxidisation in an oxygen-rich environment represent a key issue. Although some reports introduce multilayer coatings to isolate the MXene, the complex process, high cost, and relatively high thickness severely restrict the practical applications. In addition, the high cost for the fabrication of MXene is a concern. Based on the above summary and analysis, the development of MXene-based flame retardant and electrically conductive polymer coatings still has a long way to go. Although many sophisticated approaches have been developed and mechanisms have been proposed as well, several challenges still need to be addressed and overcome.

## Figures and Tables

**Table 1:** Summary of the modified MXene research work.

Materials	Modified Methods	Other Materials/Fillers	Reference
Ni flower + MXene	Mix + dip-coat	MF, PDA	[54]
CNC + MXene	Mix + LbL	Wood, PDA, APP	[58]
MXene	Anneal + LbL	Cellulose paper, AgNFs, AgNWs	[59]
Ag_2_Se NW + MXene	Mix + dip-coat	PI-aerogel, PDMS	[57]
GO + MXene	Mix + dip-coat	Cellulose paper, PA	[60]
TA + MXene	Mix + dip-coat	Cotton, PA, PANI, CNC	[56]
PDA + MXene	Mix + dip-coat	Cotton, PDMS	[55]

**Table 2:** Summary of fire safety and electrical conductivity properties of selected research works.

Coating Materials	Fire Safety Properties					Electrical Conductivity	Reference
	Cone			LOI	UL-94		
	pHRR Reductions (%)	THR Reductions (%)	TSR Reduction (%)	%	Rating	Coefficient (S/cm)	
MXene	/	/	/	/	/	2.30	[30]
MXene	44.97	19.43	/	/	/	2.40 ^#^	[31]
MXene	78.00	/	/	50	/	0.17	[33]
PPy-CS + MXene	43.03	42.40	42.42	26.80	/	/	[38]
AgNWs + MXene	31.58 *	9.09 *	/	/	/	186.00	[43]
PEI/APP + MXene + PCL	74.30	48.40	/	36.50	/	9.20	[44]
PEI/APP + MXene	/	/	/	36.50	/	6.70	[45]
OAPOSS + MXene	50.19	10.00	59.00	/	/	/	[46]
MMT/UPC + MXene/UPC	23.33 *	/	/	40.00	V-0	/	[48]
PEI + APP + PEI + MXene	42.77	/	/	39.50	V-0	777.00	[49]
CCS + MXene	66.00	/	/	45.50	/	0.20	[50]
PEI + MXene	34.39	34.88	/	/	/	11.75 ^#^	[51]
Fe_3_O_4_ nanoparticles + MXene	11.00	1.61	/	30.00	Pass	1.90	[52]
TA/CaCl_2_ + MXene	84.65	74.00	/	35.30	/	/	[53]
PA/PANI/CNC + TA/MXene	63.00	31.42	98.29	32.00	/	1.42	[56]
MXene/Ag_2_SeNWs + PDMS	66.07	/	/	53.00	/	/	[57]
PDA + APP +CNC/MXene	41.73	19.02	33.23	47.40	V-0	/	[58]

* Estimated from figures. ^#^ The unit of this value is Ω/sq.
